# Electricity and the Methods of Its Employment, Etc.

**Published:** 1889-06

**Authors:** 


ELECTRICITY AND THE METHODS OF ITS EMPLOYMENT IN REMOVING SUPERFLUOUS HAIR AND OTHER FACIAL BLEMISHES. By Plym S. Hayes, M. D. Pages, 128. Price, $1.00. Chicago: W. T. Keener. 1889.

The subject-matter of this brochure is fully described by its title. Electrolysis has been used for many years for the removal of superfluous hair, and in many cases successfully so. Dr. Hayes describes the operation, the necessary apparatus, etc., and lays all the blame for unsuccessful cases upon the unskillfulness of the operator!

